# Age and Gender: Affecting the Positive Rates of Serum PAB and ANCA in Patients with Inflammatory Bowel Disease

**DOI:** 10.1155/2021/4963641

**Published:** 2021-08-04

**Authors:** Qingquan Chen, Shirong Huang, Yue Wu, Shuyu Zhang, Qicai Liu, Min Chen

**Affiliations:** ^1^Department of Laboratory Medicine, Fujian Medical University, Fuzhou, Fujian 350004, China; ^2^Center for Reproductive Medicine, 1st Affiliated Hospital, Fujian Medical University, Fuzhou 350004, China

## Abstract

Inflammatory bowel disease (IBD) is a group of immune-mediated conditions. Immune activity is varied by age and gender. The present study is aimed at investigating the effect of age and gender on the positive rates of anti-Saccharomyces cerevisiae antibodies (ASCA), anti-neutrophil cytoplasmic antibodies (ANCA), anti-intestinal goblet cell antibodies (GAB), and antibodies to exocrine pancreas (PAB) in IBD patients. A total of 1871 hospitalized patients with confirmed IBD were included in this study. Sera were obtained from each subject for antibody measurement by indirect immunofluorescence assay. The positive rates of ANCA IgG and IgA were higher in female patients than those in male patients (*P* < 0.001) while the positive rate of PAB IgG was just reversed (*P* < 0.001). Moreover, the median ages of patients with positive ANCA IgG and IgA were higher than patients with negative antibodies (*P* = 0.0019 and *P* = 0.0110, respectively), while the median ages of patients with positive PAB IgG and IgA were significantly lower than patients with negative PAB (*P* < 0.0001). The serum levels of ANCA IgG and IgA were potentiated in old female patients, while serum PAB IgG was easy to be detected in the young male patients with IBD.

## 1. Introduction

Inflammatory bowel disease (IBD) mainly comprises Crohn's disease (CD) and ulcerative colitis (UC). It is a group of progressive immune-mediated conditions characterized by chronic inflammation of bowel and usually with a long-term treatment and unpredictable course [1, 2]. The incidence and prevalence of IBD are increasing globally, and the prevalence was more than 0.3% of the total population in many developed countries [3, 4]. The prolonged chronic inflammation also increases chance of developing colorectal cancers [5]. The diagnosis of IBD mainly depends on clinical manifestations, endoscopy, imaging, histology, and biochemistry detection. Increasing evidences show that anti-*Saccharomyces cerevisiae* antibodies (ASCA), anti-neutrophil cytoplasmic antibodies (ANCA), anti-intestinal goblet cell antibodies (GAB), and antibodies to exocrine pancreas (PAB) play a role in assisting diagnosis of IBD, especially in differential diagnosis of UC and CD [6, 7].

ASCA is directed against the oligomannosidic epitopes of *Saccharomyces cerevisiae* wall [8] and was elevated in the serum of CD patients [9]. ANCA is detected in 40%–80% of UC patients and in 6%–20% of CD patients [10]. Meanwhile, ASCA and ANCA were widely combined to serve as valuable serological tools for differential diagnosis of UC and CD [11, 12]. Similarly, PAB was directed against pancreatic antigens and was found in 30% to 40% of patients with CD. PAB have also been nominated as serological diagnostic markers for CD, though it was not correlated with clinical features of CD [13–15]. GAB was directed against goblet cells in the intestine and was found in up to 30% of patients with UC [15, 16].

Most studies to date have focused solely on the positive rates of these antibodies for the differentiation of CD from UC. Desplat-Jégo et al. [13] reported that CD patients in children who are under 15 years old displayed a higher positive rate of ASCA than in adults (mean age: 31 years), while in adults, patients under 20 years also exhibited a higher frequency of serum ASCA than patients over 20 years old in France. Similar results had also been found in the study which reported that the positive rate of PAB was higher in adult patients less than 20 years old compared to patients over 20 years old [13]. Authors also stated that CD patients more than 35 years of age were significantly less likely to express PAB, though sex was not a significant factor in PAB or GAB expression [6]. It seems that the positive rates of serum ASCA and PAB are high when the age of onset is young, or the positive rates of serum ASCA and PAB are related to the age of onset of CD, not the sex of the patients.

However, the effect of age and gender on the positive rates of ASCA, ANCA, GAB, and PAB in the IBD patients remains obscure. Herein, we investigated the relationship between age, gender, and positive rates of ASCA, ANCA, GAB, and PAB in IBD patients with large simple size and determined the effect of age and gender on the positive rates of these antibodies in IBD patients.

## 2. Materials and Methods

### 2.1. Study Subjects

This study was conducted at the 1st Affiliated Hospital of Fujian Medical University, from January 2015 to December 2019. The protocols for the study and informed consents were approved by the Fujian Medical University ethics committee (Approval number: 201536). A total of 1871 hospitalized patients with confirmed IBD were included in this study.

The inclusion criteria of the participants are as follows: (1) participants were admitted to hospital, (2) participants were diagnosed with IBD according to the Consensus on diagnosis and treatment of inflammatory bowel disease (Guangzhou, 2012) [17], and (3) written informed consents were filled correctly.

The exclusion criteria were as follows: (1) patients with gastrointestinal tumor; (2) patients with other gastrointestinal diseases, such as irritable bowel syndrome, ischemic bowel disease, intestinal polyps, intestinal vascular malformations, and eosinophilic gastroenteritis; (3) patients with bacterial or viral infection; and (4) patients with other autoimmune diseases.

### 2.2. Antibody Measurement

Venous blood 2 mL was obtained from each subject. Sera were separated immediately and kept at -80°C before analysis. IgA and IgG of ASCA, ANCA, GAB, and PAB were detected by indirect immunofluorescence (IIF) using commercially available detection kits (EUROIMMUN Medical Diagnostics Co., Ltd.) according to the manufacturer's instructions. In brief, sera were diluted 1 : 10 in phosphate buffer. 25 mL of the diluted sera was incubated for 30 min on slides with smears of Saccharomyces cerevisiae, ethanol-fixed human neutrophil, monkey small intestine, and monkey pancreas for ASCA, ANCA, GAB, and PAB, respectively. After a washing step, fluorescent-conjugated goat anti-human IgG or IgA was added to detect IgG or IgA of these antibodies, respectively.

### 2.3. Statistical Analysis

All data were analyzed with SPSS version 18.0 (SPSS Inc., Chicago, IL, USA). Graphs were plotted with the GraphPad Prism 5 software. Continuous variables were expressed as median (interquartile range) and calculated by Mann–Whitney test. Categorical variables were expressed as percentage and compared by *χ*^2^ test for unpaired data and McNemar's test for paired data between groups. *P* < 0.05 was considered statistically significant.

## 3. Results

### 3.1. Presenting Characteristics

The study cohort included 1871 hospitalized patients with confirmed IBD. The median age was 39 years (IQR, 26-55 years), and 757 (40.5%) were women.

### 3.2. Positive Rates of ASCA, GAB, PAB, and ANCA in Patients with IBD

**Figure 1:**
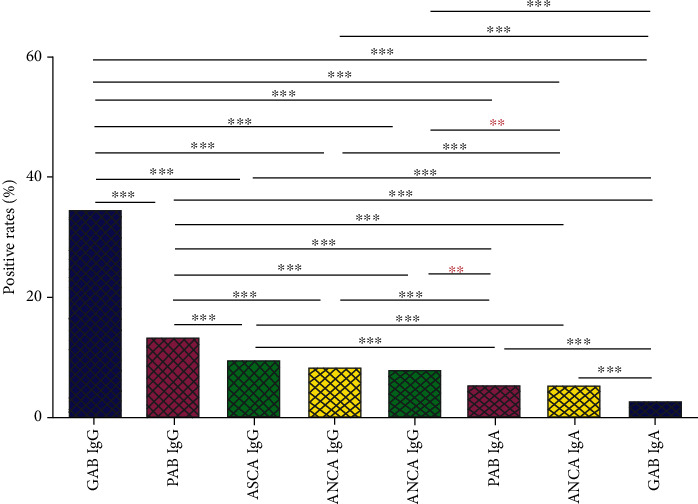
Positive rate of ASCA, GAB, PAB, and ANCA in patients with IBD. Positive rate was presented as percentage and calculated by using McNemar's test. ASCA: anti-*Saccharomyces cerevisiae* antibodies; GAB: anti-intestinal goblet cell antibodies; PAB: antibodies to exocrine pancreas; ANCA: anti-neutrophil cytoplasmic antibodies; IBD: inflammatory bowel disease; ^∗∗∗^*P* < 0.0001; ^∗∗^*P* < 0.001.

To assess the positive rates of antibodies known to be associated with IBD, the antibodies including ASCA IgG, ASCA IgA, GAB IgG, GAB IgA, PAB IgG, PAB IgA, ANCA IgG, and ANCA IgA were measured (Table 1 and Figure 1). Our results showed that the positive rate of GAB IgG was 34.31%. It was the highest among these antibodies (all *P* < 0.001). Next to GAB IgG, the positive rate of PAB IgG was 13.04%, which was the second highest among these antibodies. And the positive rate of other antibodies ranged from 2.57% to 9.30%. Moreover, the positive rates of ANCA IgG and ANCA IgA were higher in female patients than those in male patients (10.83% vs. 6.55%, *P* < 0.001 and 7.27% vs. 3.68%, *P* < 0.001, respectively), while the positive rate of PAB IgG was significantly lower in female patients than that in male patients (9.78% vs. 15.26%, *P* < 0.001). No distinguishing positive rates of other antibodies were observed in female patients and as compared with male patients with IBD.

### 3.3. Ages of ASCA, GAB, PAB, and ANCA in Patients with IBD

To further evaluate the characteristics of age distribution in patients with IBD, the ages of patients with and without the antibodies, together with the ages of female and male patients with the positive antibodies, were analyzed. For ASCA IgA, ANCA IgG, and ANCA IgA, the median ages of patients with these antibodies were higher than those of patients without these antibodies (43.0 vs. 39.0, *P* = 0.0203; 45.0 vs. 39.0, *P* = 0.0019; and 44.5 vs. 39.0, *P* = 0.0110, respectively), while the median ages of patients with PAB IgG and PAB IgA positive were significantly lower than those of patients without PAB IgG and PAB IgA (27.5 vs. 42.0, *P* < 0.0001 and 26.0 vs. 40.0, *P* < 0.0001, respectively). There was no significant difference in the median ages between antibody-positive patients and antibody-negative patients for ASCA IgG, GAB IgG, and GAB IgA (Table 2).

Interestingly, there was a higher median age in the female patients compared to male patients with positive ASCA IgA (52.0 vs. 40.0, *P* = 0.0354), while there was no significant difference in the median ages between female and male patients with the other positive antibodies (Table 3).

## 4. Discussion

In this study, we investigated the relationship between age, gender, and the positive rates of ASCA, ANCA, GAB, and PAB in IBD patients. Our study found that GAB IgG had a positive rate of 34.31%, which was the highest positive rate among these antibodies in IBD patients. Interestingly, the positive rates of ANCA IgG and IgA were higher in female patients than those in male patients while the positive rate of PAB IgG was significantly lower in female patients than that in male patients. Moreover, the median ages of patients with positive ASCA IgA, ANCA IgG, and ANCA IgA were higher than patients with negative antibodies, while the median ages of patients with positive PAB IgG and IgA were significantly lower than patients with negative PAB.

The intestinal infection, disorder of immune regulation in the intestinal mucosal, and gene susceptibility are main factors related to IBD [18]; therefore, serological antibodies, especially ASCA, ANCA, GAB, and PAB, were often measured to aid diagnosis of IBD or distinguish CD from UC. Unfortunately, the expressions of these antibodies are not high. For patients with CD, ASCA was positive in approximately 40% to 54%, while PAB was detected in 30% to 46% of patients [6, 7, 13]. And for patients with UC, ANCA was positive in approximately 50% to 75%, while GAB was detectable in 2%-46.4% of patients [6, 7, 13]. In line with these studies, our results showed that the positive rate of GAB IgG was 34.31%. And the expressions of PAB, ASCA, and ANCA were between 2.57% and 13.04% in our study, which was not as high as previous reported [6, 13]. One possible reason for this difference was that the positive rates of these antibodies were from all the patients including CD and UC patients. The expression rates of these antibodies for CD patients or UC patients only were not calculated. In addition, the expression rates of these antibodies may be related to different ethnic group. A similar finding also showed that the positive rates of ANCA, ASCA, and PAB were significantly different between Chinese and Caucasian patients with IBD [6].

In our study, pancreatic antibody PAB was also detected in the patients with IBD, and the positive rate was 13.04%. PAB was also measured in the patients with IBD by Desplat-Jégo et al. [13] and Lawrance et al. [6];Vimal Bodiwala, Timothy Marshall, Kiron M Das, Steven R Brant, Darren N Seril they reported that PAB expression was highly specific for CD, though it was without any correlation with clinical characteristics of the disease including whether the patients have pancreatitis or not. So, there is no evidence for a direct pathogenic role for PAB in CD. Patients with IBD often have pancreatic diseases such as acute pancreatitis, chronic pancreatitis, or pancreatic exocrine dysfunction [19], which may lead to pancreatic antigen release from pancreas and stimulate the production of pancreatic antibodies. IBD is a group of immune-mediated conditions, and autoantibodies including pancreatic antibodies may be exhibited in the abnormal activation of immune system. These mechanisms may partially explain the presence of PAB in IBD patients with pancreatic diseases. But why did PAB exhibit in the IBD patients without pancreatic disease? Maybe the generation of PAB is the result of a cross-reactivity with enteric microbial antigens [20], which has recently been demonstrated for ANCA in UC [21]. Maybe the pancreas like antigens was expressed in the regional intestine under the chronic inflammation of the intestine. Anyway, up until now, a mechanism association between PAB and CD had not been demonstrated. Therefore, the mechanisms of PAB appeared in the IBD patients need to be further studied in the future.

Growing evidences indicate that autoimmunity is influenced by gender. The immunoreactivity including antigen presenting activity and mitogenic responses of lymphocytes and monocytes in females is more enhanced in females than in males. The immunoglobulin levels in females are also higher than males, due to the influence of sex hormones and sex related genes [22]. And IBD should be regarded as an autoimmune disease because of the reactivity of lymphocytes to their own antigens [23] and the autoimmune extraintestinal manifestations [24]. Consistent with these theories, our study found that the positive rates of ANCA IgG and ANCA IgA were higher in female patients than in male patients. Our results are also agreed with Hornig et al. [25] who reported that the level of serum antinuclear was increased in women than in men. Contradictory to those studies [22, 25], the positive rate of PAB IgG was significantly lower in female patients compared to male patients. And we failed to see a significant difference between female and male patients for the positive rates of ASCA and GAB. Therefore, our study reflected that gender can affect the expressions of autoantibodies, and some of which were highly expressed in women while some of which are highly expressed in men.

Age may also be related to the positive rates of autoantibodies. And elderly patients have a relative immunodeficiency compared to younger patients [26]. In our study, we found that the median ages of patients with ASCA IgA, ANCA IgG, and ANCA IgA were higher than patients without these antibodies. Our results were similar with the study conducted in USA which reported that cystic fibrosis patients who were ASCA seropositive were older than the seronegative patients [27]. But our results were partly contrasted with Desplat-Jégo et al. [13] who documented that young CD patients (under 15 years old in children or under 20 years old in adults) had a higher ASCA seropositivity than old patients in France. These discrepancies probably relate to distinct racial and ethnic groups [28] and dietary habits or environmental exposures, because bread is a great source of *Saccharomyces cerevisiae* and is also a major source of gluten which was usually eaten in France [13]. Consistent with previous studies [6, 13], our results also found that the median ages of patients with PAB IgG and PAB IgA positive were significantly lower than patients without PAB IgG and PAB IgA. Additionally, a higher median age in the female patients with positive ASCA IgA was also found in our study. Therefore, our study suggests that age may also have influence on autoantibodies.

This study had some limitations. First, we did not access the influences of age and gender on the expression of autoantibodies in CD and UC subgroups. Second, how age and gender affect the expression of these autoantibodies was not included in the study.

## 5. Conclusion

Age and gender can affect the expressions of antoantibodies in patients with IBD. The levels of ANCA IgG and IgA were potentiated in old female patients, while the level of PAB IgG was easy to be detected in the young male patients. Our study inflects that the influence of age and gender on the results of autoantibodies should be considered in the clinical application.

## Figures and Tables

**Table 1:** Positive rate of ASCA, GAB, PAB, and ANCA in patients with IBD.

Antibodies	No. (%)			*P* value
	Total (*N* = 1871)	Female (*n* = 757)	Male (*n* = 1114)	
ASCA IgG	149 (7.96)	71 (9.38)	78 (7.00)	0.062
ASCA IgA	174 (9.30)	65 (8.59)	109 (9.78)	0.381
GAB IgG	642(34.31)	249 (32.89)	393 (35.28)	0.286
GAB IgA	48 (2.57)	21 (2.77)	27 (2.42)	0.638
PAB IgG	244 (13.04)	74 (9.78)	170 (15.26)	<0.001
PAB IgA	97 (5.18)	39 (5.15)	58 (5.21)	0.958
ANCA IgG	155 (8.28)	82 (10.83)	73 (6.55)	<0.001
ANCA IgA	96 (5.13)	55 (7.27)	41 (3.68)	<0.001

Data were presented as No. (percentage) and calculated by using *χ*^2^ test. ASCA: anti-*Saccharomyces cerevisiae* antibodies; GAB: anti-intestinal goblet cell antibodies; PAB: antibodies to exocrine pancreas; ANCA: anti-neutrophil cytoplasmic antibodies; IBD: inflammatory bowel disease. *P* values indicate differences between female and male patients with IBD. *P* < 0.05 was considered statistically significant.

**Table 2:** Ages of ASCA, GAB, PAB, and ANCA in patients with IBD.

Antibodies	Median (IQR), years		*P* value
	Positive	Negative	
ASCA IgG	40.0 (25.0-63.0)	39.0 (26.0-54.0)	0.0615
ASCA IgA	43.0 (23.8-64.3)	39.0 (26.0-53.0)	0.0203
GAB IgG	37.0 (26.8-53.0)	41.0 (25.0-56.0)	0.3446
GAB IgA	31.5 (23.3-49.8)	39.0 (26.0-55.0)	0.0887
PAB IgG	27.5 (22.0-34.0)	42.0 (27.0-56.0)	<0.0001
PAB IgA	26.0 (20.0-33.0)	40.0 (26.0-55.3)	<0.0001
ANCA IgG	45.0 (31.0-57.0)	39.0 (25.0-54.0)	0.0019
ANCA IgA	44.5 (31.3-55.8)	39.0 (25.0-55.0)	0.0110

Data were presented as median (IQR) and calculated by Mann–Whitney test. IQR: interquartile range; ASCA: anti-*Saccharomyces cerevisiae* antibodies; GAB: anti-intestinal goblet cell antibodies; PAB: antibodies to exocrine pancreas; ANCA: anti-neutrophil cytoplasmic antibodies; IBD: inflammatory bowel disease. *P* values indicate differences between antibody positive and negative patients. *P* < 0.05 was considered statistically significant.

**Table 3:** Ages of patients with positive ASCA, GAB, PAB, and ANCA.

Antibodies	Median (IQR), y		*P* value
	Female	Male	
ASCA IgG	47.0 (27.0-65.0)	35.0 (23.0-62.0)	0.1099
ASCA IgA	52.0 (27.5-66.5)	40.0 (21.0-61.0)	0.0354
GAB IgG	38.0 (27.0-53.5)	36.0 (26.0-52.5)	0.2218
GAB IgA	32.0 (25.0-47.5)	31.0 (22.0-50.0)	0.7949
PAB IgG	27.5 (22.0-37.3)	27.5 (21.0-34.0)	0.3483
PAB IgA	25.0 (19.0-36.0)	28.0 (20.0-32.0)	0.9736
ANCA IgG	48.0 (33.3-58.8)	40.0 (29.0-52.5)	0.0728
ANCA IgA	47.0 (33.5-61.0)	40.0 (29.0-52.5)	0.1035

Data were expressed as median (IQR) and calculated by Mann–Whitney test. IQR: interquartile range; ASCA: anti-*Saccharomyces cerevisiae* antibodies; GAB: anti-intestinal goblet cell antibodies; PAB: antibodies to exocrine pancreas; ANCA: anti-neutrophil cytoplasmic antibodies; IBD: inflammatory bowel disease. *P* values indicate differences between female and male patients. *P* < 0.05 was considered statistically significant.

## Data Availability

The data used to support the findings of this study are currently under embargo while the research findings are commercialized. Requests for data, 12 months after publication of this article, will be considered by the corresponding author.
